# Learning what our target audiences think and do: extending segmentation to all four bases

**DOI:** 10.1186/s12889-019-6696-2

**Published:** 2019-04-05

**Authors:** Anna Kitunen, Sharyn Rundle-Thiele, Mohammad Kadir, Abi Badejo, George Zdanowicz, Megan Price

**Affiliations:** 1 Social Marketing @ Griffith, Griffith University 170 Kessels Road Nathan, Qld, Brisbane, 4111 Australia; 2 Enhance Research, 30 Misterton Street Fortitude Valley Qld, Brisbane, 4006 Australia; 3 Queensland Health, 33 Charlotte Street, Brisbane, Queensland 4000 Australia

**Keywords:** Social marketing, Behaviour change, Segmentation, Two-step cluster analysis, Sexual health, Young people

## Abstract

**Background:**

While acknowledged as one of social marketing’s necessities, limited reporting of segmentation exists. The current study seeks to extend segmentation drawing on all four segmentation bases within the context of Queensland young adult sexual health behaviour.

**Methods:**

An online survey was used to collect data from 15 to 29 year old people in Queensland, Australia. Data collection was undertaken online to capture the broader population of young adults and in person on campuses to gather data from students who were currently enrolled at University. Quotas were set to ensure a broad representation was attained reflecting the States demography.

**Results:**

Two-step cluster analysis revealed three different segments. The most important variables in segment formation were age, household type, experience of risky sexual encounters and previously being tested or treated for sexually transmissible infections (STIs). The results suggest that demographic and behavioural variables were the most effective in segment definition.

**Conclusions:**

This study investigated young people aged 15–29 in Queensland, Australia to examine group differences drawing from four bases. This study revealed three distinct segments in a sexual health context and highlighted the importance of behavioural variables in segment formation, insight and understanding.

**Electronic supplementary material:**

The online version of this article (10.1186/s12889-019-6696-2) contains supplementary material, which is available to authorized users.

## Background

Sexually transmitted infections (STIs) are a long-term public health problem that can lead to severe complications including pelvic inflammatory disease, infertility and ectopic pregnancy [1, 2]. Unprotected sex can lead to acquisition and transmission of STIs [3]. STI rates among young people continue to rise [4–9] and research suggests that while young people possess greater knowledge of the risk of STIs, they have a lower knowledge of correct safe sex behaviour [10] with under 25 year olds being at greatest risk [3]. STIs are prevalent among young Australians with higher STI notification rates for people aged 15–29 [11]. The increasing rates of STI notifications for Australians is comparable with data from the United States, New Zealand, UK and Canada [12–15]. The growing number of STIs among young people is associated with frequently changing sexual partners and engaging in high risk sexual behaviours [16]. The risk of negative health behaviour rises on university campuses where parental oversight is restricted [17]. Current literature provides insights into sexual behaviours and STI awareness among young people in Australia and abroad however, current data on group differences within at risk populations is limited. Peer crowd studies show promise for improving health promotion and practice via targeting [18]. Young people identify to their peer environment by relating with these crowds and tend to consider belonging to widely acknowledged and categorised social types along with others [19]. Research shows that peer crowd identification is linked with risk behaviours suggesting that these crowds represent social types with prominent health behaviour norms [20]. Understanding group differences might enable the development of sexual health programs and interventions to encourage positive health behaviours such as STI testing [21]. There is a need for effective methods to change negative sexual health behaviours and increase STI awareness in order to increase positive health behaviours.

### Social marketing

An approach that can help to promote a change in sexual health behaviours is social marketing [22]. Social marketing was first introduced nearly 50 years ago proposing that marketing concepts could be applied to promote social causes [23]. This led to the first definition of social marketing:

“*Social marketing is the design, implementation, and control of programs calculated to influence the acceptability of product planning, pricing, communication, distribution and marketing research*”.

Social marketing incorporates commercial marketing techniques in the planning, analysis, implementation and assessment of programs designed to influence target audiences to engage in voluntary behaviour change [24–26]. Andreasen [27] created six social marketing benchmarks to differentiate social marketing from other health and behavioural change sciences. The six social marketing benchmarks put forward by Andreasen include behaviour change, exchange, formative research, segmentation, marketing mix and competition [27]. Studies show that when more benchmarks elements are correctly applied, behaviour change is more likely [28, 29]. However, a recent umbrella review summarises the limited reported use of segmentation in available peer reviewed literature [30]. Examination of systematic reviews extending beyond the umbrella review provide further evidence of the lack of reported segmentation use in scholarly literature [31, 32]. In the rare cases where segmentation is reported the most frequently used bases are demographic and geographic [33], despite the availability of other segmentation bases [34]. Recent research shows that health and social marketing campaigns targeting high risk subgroups within a health disparity population can benefit from targeting different groups by developing more effective and efficient campaigns [18].

The current study draws on the social marketing benchmark of segmentation. Segmentation theory and its use in social marketing contexts guarantees further examination to understand if segments can be identified on all four segmentation bases (demographic, geographic, psychographic, behavioural) in order to gain consumer insights to contribute in the planning, analysis, execution and evaluation of social marketing interventions to influence sexual health behaviour. Therefore, the aim of this study is to two-fold: first, to demonstrate how segmentation using two-step cluster analysis can be used to find segments in a sexual health context using all four segmentation bases, and second, utilising identified segments to offer practical implications for social marketers working in the sexual health area.

### Segmentation

Segmentation is considered as a key benchmark criterion for effective social marketing practice by many authors who have included segmentation in their social marketing frameworks [27, 35, 36]. Segmentation comprises of three main parts: first, finding homogenous segments within a bigger heterogeneous group, second, assessing and choosing one or several segment(s), and finally developing a program, service or communication strategy matched to the target segment(s) unique needs, wants and characteristics [37]. Segmentation is widely used in commercial contexts [38] and segmentation has been identified as an effective way to target messages to reach specific groups delivering attitude and behaviour change in social marketing context [39]. Segmentation also provides social marketers with additional insight to understand the groups that are most likely to change and offers various benefits including a more nuanced understanding of the market given different group interests are accommodated, which in turn enhances ability to more precisely predict behavioural change and increases the likelihood of identifying and utilising a broader array of market opportunities [40].

Researchers have previously segmented target audiences based on their demographic and geographic bases in order to find indicators that predict behaviour [33, 41, 42]. Drawing on wider segmentation literature, researchers [43–45] have criticised demographic and geographic segmentation bases for their failure to predict behaviour (e.g. use a condom). A key explanation for the frequent use of demographic and geographic bases may lie in other studies [46]. For example, one study acknowledged that demographic and geographic variables, unlike psychographic variables, are directly observable and therefore offer assistance in program planning and design. For example, demographic and geographic variables assist marketing managers to decide where to best allocate limited budgets. While demographic variables are not able to predict which people will perform a behaviour they will continue to play an important role in program planning and design given they can be directly linked to understanding where to locate programs and services and which communication channels to use.

Analyses focussing on demographic and geographic variables restrict insights into the types of people (education status, gender, income levels) and the places lived; all of which are directly observable. Limiting segmentation insights into demographic and geographic measures prevents an understanding of how and why the target audience currently behaves from emerging. Acknowledging the potential to develop a more explanatory and predictive approach, psychographic and behavioural variables have recently been incorporated in segmentation analysis [26, 44, 47–49]. For example, a recent study indicates the importance of psychographic and behavioural variables stating that these variables were the most important measures in segment formation based on the predictor importance score while demographics were less important [47]. This finding is supported in an active school travel context where psychographic and behavioural variables were once again key in segment formation [50]. Delivering insights into what people think and understand about how and why current behaviours occur, offers the potential to improve prediction of behaviours in addition to extending insights that can be used during program planning and design. The inclusion of psychographics and behaviours in segmentation formation delivers information on behaviours performed and the underlying psychological and social reasons potentially driving the behaviour in addition to observable demographic and geographic variables.

Taken together, a review of previous peer reviewed literature indicates limited application of segmentation in health prevention and behavioural change research more broadly. Moreover, limited research attention has been directed towards psychographic and behavioural segmentation bases. Researchers [46] suggest the use of combined segmentation variables (demographics, psychographics, geographic and behavioural variables) is warranted. The omission of psychographic and behavioural bases restricts the insights that can be gained by to inform sexual health program planning and implementation. A key gap in understanding may be addressed through the use of all four segmentation bases. To date, only a few studies have utilised all four segmentation bases [49, 51, 52]. Therefore, the purpose of this study is to report whether segments can be formed on the basis of all four segmentation bases in a sexual health context. Consistent with prior research [50] the two-step cluster analysis method is employed to assess measures capturing all four segmentation bases, namely demographics (age, household type), psychographic (STI understanding, self-efficacy), behaviour (risky sexual encounters, tested or treated for STIs) and geographic (region). Additionally, this study demonstrates how additional information collected can be reported by segment providing a rich dashboard of information to inform program design and planning.

## Methods

This study used a convenience, cross-sectional sample located in one Australian state, namely Queensland. The target population for this study were young people aged between 15 and 29 years old. As previously stated, this study employed measures representing all of the four segmentation bases, namely demographic, geographic, psychographic and behavioural variables. Measures assessed in this study included previously used sexual health behaviour measures sourced from the literature, namely, experience of risky sexual encounters [9], being tested or treated for STIs [8], knowledge and understanding of STIs [53] and self-efficacy [54]. Other measures included were age, household type and region. All measures employed in cluster analysis exhibited zero to low correlations (*r* > 0.3) as recommended by Dolnicar et al. [55]. Following cross-sectional research design, an online survey of approximately 15 min in length was utilised to collect data from university students located at South East Queensland and non-university students across the state and in metro and regional areas of Queensland, Australia. An additional file shows the survey in detail (see Additional file 1).

University student sample was recruited via broadcast email including research project description and link to the survey, and also in person on campuses to collect data from students who were currently enrolled at University. Participants were offered an equal chance of winning one of ten $50 vouchers and coffee vouchers valued at $3.80 each for students completing the survey on campus to incentivise their participation in line with the recommendations of Dillman et al. [56]. Following the ethical approval central administration of 10 Queensland universities were contacted; five universities agreed to support and assist data collection and data was received from three South East Queensland universities (33% university response rate). In total, 458 students responded to the study online, while 268 were intercepted across two university campuses between August 3 and 17, 2017. To ensure a broad cross section of 15–29 year olds two panel providers (Q&A and Student Edge) were used to recruit a profile to ensure coverage of the entire state including sufficient representation across both metro and regional areas. A total of 1451 surveys were collected.

Knowledge and understanding of STIs and self-efficacy were measured using an eleven-point Likert scale (0 = Strongly Disagree and 10 = Strongly Agree) with an additional option for “Not Applicable/Don’t know”. Knowledge and understanding of STIs [53] (*a* = 0.91) and self-efficacy (*a* = 0.92) measures showed good internal reliability exceeding the recommended levels. Self-efficacy items were sourced from Shaweno et al. [57] and included statements such as “I feel confident discussing condom usage with a partner”, “I am confident that I would use a condom during sex even after drinking alcohol” and “I would insist on using condoms during sex, even if my partner didn’t want to.” Respondents were asked in Yes/No format to report risky sexual health and STI testing and treatment behaviours. Risky sexual health behaviours which were summed into an index (0–7) included reporting of anal sex, anonymous sex, casual sex, group sex (3 or more people), having sex while drunk, having sex while high and having sex with someone via a hook up app [9]. STI testing and treatment was summed into an index (0–2) for reports of ever being tested for STI and ever being treated for an STI [8].

Power analysis was conducted on the basis of the F-test – ANOVA: Fixed effects, omnibus, one-way, using G*Power software 3.0.10 [58], which was especially developed for sample size calculation. Sample size was calculated with 80% power and *p* < 0.01 for 0.10 effect size. Protocol of power analysis reported that 1395 participants were needed to detect differences among segments.

After data collection and data cleaning, two-step cluster analysis was employed with IBM SPSS Statistics 25 to identify homogenous subgroups in the population. Two-step cluster analysis is considered the most appropriate technique for this study due its exploratory nature, particularly since the number and the members of the clusters are unknown [59]. Additionally, two-step cluster analysis forms segments based on both categorical and continuous data [60] offering an ability to simultaneously consider a wide and diverse range of measures and it is able to process large sample sizes [61]. Two-step cluster analysis has been previously used to identify segments in adolescent populations [44], tourism segments [62], to explore health behaviours [63] and coping with psychological stress [64]. The analysis was implemented using 7 segmentation variables. The measure to respondent ratio was within recommended guidelines [65], exceeding the recommended 1 to 100 ratio. First, original cases were grouped into pre-clusters based on log-likelihood distance [66]. This was then reduced to the best number of clusters based on the Schwartz’s Bayesian information criterion (BIC) [60]. Segments were validated using a split sample to ensure the cluster formation was consistent in a half sized sample. Chi-square and One-way ANOVA tests were conducted on the categorical and continuous variables to examine group differences.

## Results

Two-step cluster analysis produced a sample (*n* = 1451) with a silhouette measure of cohesion and separation of 0.2. A silhouette measure of cohesion and separation of more than 0.0 is needed for the within-cluster distance and the between cluster distance to be valid [60]. Three segments were identified (see Table 1) and a segment solution with seven segmentation variables was confirmed as the final solution. After validation of segments, chi-square tests were conducted on categorical variables with statistical differences noted for the seven variables included with the segment model. ANOVA tests were conducted on continuous variables (e.g. tested or treated for STIs and risk sex behaviours), revealing significant differences between segments based on all of the continuous variables.

**Table 1:** Summary of two-step cluster analysis results

Segmentation variable	Importance	Sexually experienced *n* = 626 (43.1%)	Climbing the sexual ladder *n* = 423 (29.2%)	STI unawares *n* = 402 (27.7%)	Significance
Age	1.00	26 to 29 (47.6%)	18 to 21 (100%)	15 to 17 (100%)	.000*
Household type	0.53	Live with partner (27.3%)	Live home with parents (57%)	Live home with parents (98.8%)	.000*
Risky sexual encounter	0.16	2.2 (2.1)	1.3 (1.8)	0.5 (1.1)	.000*
Tested or treated for STIs	0.14	0.6 (0.7)	0.3 (0.6)	0.1 (0.3)	.000*
Region	0.06	City (78%)	City (97.2%)	City (74.6%)	.000*
STI understanding	0.04	6.5 (2.0)	6.0 (2.2)	5.5 (2.1)	.000*
Self-efficacy	0.04	7.6 (2.2)	7.9 (2.0)	8.5 (1.6)	.000*

Note *p < .001

Two variables were found to be highly important to cluster formation in this study: age (Importance = 1.00) and the type of household where respondents live (Importance = 0.53). Also respondents experience of risky sexual encounters (Importance = 0.16) and ever being tested or treated for STIs (Importance = 0.14) were relatively important in cluster formation. Each segment will now be discussed in turn drawing on wider available research data to derive a detailed understanding of the identified segment (see Table 2).

**Table 2:** Three segment solution – demographic & geographic variables

	Total 100% *n* = 1451	Sexually experienced 43.1% *n* = 626	Climbing the sexual ladder 29.2% *n* = 423	STI unawares 27.7% *n* = 402	*p*
Region					.000
Inner		11.7%	2.6%	12.4%	
City		78%	97.2%	74.6%	
Outer/remote		10.4%	0.2%	12.9%	
Age					.000
15 to 17		1.3%	0%	100%	
18 to 21		5.6%	100%	0%	
22 to 25		45.5%	0%	0%	
26 to 29		47.6%	0%	0%	
Household type					.000
I live at home with my parent/s		17.7%	57%	98.8%	
I live alone		8.1%	4.7%	0%	
I live in a shared household		23.6%	19.6%	0%	
I live with my partner		27.3%	8.5%	0%	
I am a single parent		2.1%	0%	0%	
I am in a couple with children at home		18.5%	0%	0%	
I live in shared student accommodation (e.g. on campus)		1.6%	9.2%	0.7%	
Other		1%	0.9%	0.5%	

Segment one termed “*Sexually experienced*” is the largest and the oldest segment (94% are aged 22 and over). This segment is the most sexually active with 88% of respondents reporting an active status and that they have had sex with one or more partners in the past 3 months (87%). This segment is the most likely to have been tested (47%) and treated (12%) for STIs even though most of them haven’t been tested recently with almost half of this segment being tested for STIs more than one year ago (47%). However, testing is seen the norm with 35% thought that they should get tested and one in four (26%) being regular testers. This segment is the most likely of the three segments to indulge in risky sexual behaviours with 2 in 3 respondents reporting having had sex while drunk (67%) and one in five reporting anonymous sex (20%). Few respondents in this segment report knowing a lot about STIs. For example, between 10 and 32% of respondents in this segment report knowing a lot about HIV (31%), genital herpes (23%), chlamydia (21%), genital warts (12%), and other STIs. Respondents in this segments consider Internet (79%) the key source of information along with friends and peers (65%). Insights reported for segment one are consistent with Senior et al. [10] who identified that young people possess greater risk of STIs and have a lower knowledge of correct safe sex behaviour.

While respondents in this segment report the highest rates of risky sexual encounters and STI testing and treatment they report the lowest rates of self-efficacy when compared to segments two and three. Self-efficacy refers to perceptions of one’s ability to perform the behaviour [54]. Segment one, arguably the most experienced segment in terms of sex, unsafe sex, STI treatment and testing, report the lowest ability to insist on using condoms when not wanted by a partner (M = 7.4) or when things were getting intense (M = 7.3). This segment reported a lower ability to use condoms when high (M = 6.8) and when they were drunk (M = 6.8) and importantly this segment was most likely to disagree that using a condom can be fun (M = 5.7). Attitudes towards sex are largely positive with respondents reporting being comfortable with their sexuality (M = 8.4), enjoying sex (M = 8.2) and seeing sex as an expression of intimacy (M = 8.1).

The second segment – “*Climbing the sexual ladder”* (*n* = 423) are young adults aged 18–21 years old. Many in this segment are sexually active (68%) and report having sex with one or more partners in the past three months (83%). When compared to segment one, respondents in this segment have had significantly less risky sexual encounters, and they reported less STI testing and treatment. Nearly half (41%) reported having sex while drunk and one in three report having casual sex (30%). One in ten reported having anonymous sex (10%) and 16% reported having sex after using a hook up app. Respondents in this segment had a reasonably high knowledge of STIs reporting knowing a lot about HIV (34%), genital herpes (24%), chlamydia (23%) and genital warts (17%). Similarly to segment one respondents in this segment haven’t been tested recently but most of them have been tested within the past year (34%) and testing is seen as the norm with 34% thought that they should get tested and 29% were regular testers.

In comparison to segment one, segment two reported higher self-efficacy rates. Segment two, which reports less sexual experience, lower levels of unsafe sex, STI treatment and testing when compared to segment one report higher abilities to insist on using condoms when not wanted by a partner (M = 7.8) or when things were getting intense (M = 7.6). When compared to segment one this segment reported a higher ability to use condoms when high (M = 7.1) and when they were drunk (M = 7.3). When compared to segment one this segment were more likely to agree that using a condom can be fun (M = 6.3). Attitudes towards sex a largely positive but slightly less when compared to segment one. Segment two reported enjoying sex (M = 8.3), being comfortable with their sexuality (M = 8.0), being able to form respectful sexual relationships (M = 8.0) and seeing sex as an expression of intimacy (M = 7.9).

When compared to segments one and two the third segment – “*STI unawares”* are the least sexually active and they report the lowest levels of risky sexual behaviours. In segment three, majority of (42%) 15–17 year olds report being sexually active and few report having anonymous sex (3%) or sex with someone via a hook up app (3%). While majority of this segment had one partner over the past three months (71%) respondents in this segment have a moderate awareness of STIs with nearly a third reporting knowing a lot about HIV (29%) followed by genital herpes (19%), chlamydia (18%) and pubic lice (13%). Respondents in this segment haven’t been tested for STIs recently but most of them within the past three months (35%), which is more recently than the other two segments. This segment also sees testing as the norm with majority saying they thought they should get tested (42%). Of note, knowledge of STI treatment (M = 4.4) and STI testing are lowest in this group (M = 5.3%). Similar to segments one and two, segment three are most likely to use the Internet and friends as key STI information sources.

In comparison to segments one and two, segment three reported highest self-efficacy rates. Even though being the least sexually experienced and reporting the lowest levels of STI treatment and testing out of the three segments respondents report highest ability to use condoms to avoid getting STIs (M = 9.1), highest abilities to use condoms when things were getting intense (M = 8.6) or insist on using condoms when not wanted by partner (M = 8.5). Out of all of the segments this segment were most likely to agree that using a condom can be fun (M = 6.4) and that they would use condom even if it was less fun (M = 8.5).

When applied to safe sex and STIs, segmentation analysis drawing on all four segmentation bases was able to identify three meaningful segments. Insights gained from segmentation analysis indicate that sexual experience is the distinguishing factor for segments. Sexually experienced (and generally older) people know more about STIs and feel less able (lower self-efficacy) to have conversations about safe sex. In contrast, those with less sexual experience report the highest levels of confidence to have safe sex conversations and the lowest level of knowledge about STIs in general and importantly STI testing and treatment.

## Discussion

Limited research is available in peer reviewed literature reporting on segmentation practice, a core social marketing feature [27]. The aim of this study was to establish if distinct segments were evident in a sexual health context drawing from measures sourced from four segmentation bases extending application of segmentation to all recommended bases [46]. This study indicates how researchers can use two-step cluster analysis to identify segments, which are represented by a group of individuals who share similar characteristics that differ from other groups in the larger heterogeneous target audience. Further, this study demonstrates how available information can be used delivering a dashboard to inform program design and planning.

Three different segments were discovered with behavioural and demographic variables being the most important variables in cluster formation. All variables were significantly different suggesting differences in all modelled segmentation variables between the three segments. The results of this study support the use of all four bases as recommended in segmentation theory to identify segments [46]. The current study differs from most segmentation studies [67, 68] given that all four segmentation bases were used. This is an improvement on studies that use three or less segmentation bases [69, 70] or studies that do not use segmentation at all. In the past, in the rare instances where segmentation use is reported in peer reviewed social marketing studies [71], demographic and geographic variables have dominated practice [67, 68]. The addition of both psychographic and behavioural variables in this study highlighted the dominant role of sexual experience in segment formation. The combination of reported behaviours, STI understanding and self-efficacy in addition to age, provides a more holistic and nuanced view of groups (segments) and the measures driving this distinction. Consideration of multiple measures (*n* = 7 in this study) permitted knowledge, sexual and STI behaviours, attitudes towards STIs and self-efficacy to be considered along with age (demographic) and places lived (geographic). Past research extending social marketing bases and employing two-step cluster analysis has identified the importance of psychographic and behavioural variables in cluster formation. In Dietrich et al. and Schuster et al. [44, 50] psychographic and behavioural variables were the most important measures in segment formation based on the predictor importance score. The results of this study underpin the importance of continued inclusion of the demographic base based on the importance this base had along with behaviour on age. Together, these studies do indicate that the behavioural base is essential in cluster formation.

This study has important implications for social marketing and research practice. The method reported in this study, two-step cluster analysis, offers one segmentation method for social marketing. Two-step cluster analysis provides a comprehensive description of segments that can guide program development. Segmentation can be applied to better customise the design of intervention programs to cater to the needs of different segments, with the aim to increase correct safe sex behaviour and hence contribute to STI prevention. Formative research can assist in developing an appropriate social marketing mix catering to the different wants, needs and characteristics of the identified segments. For example, given identification of three segments social marketers could next embark on co-design with each segment (for an example of this approach see Dietrich et al.) [72] to ensure meaningful programs are built catering to the unique needs and wants of each segment group. Further, insights into safe sex practices of different segments can be acquired through quantitative and qualitative data to then customise better social marketing programs for different target audiences.

This study contributes to knowledge outlining an approach to segmentation, namely two-step cluster analysis applied to all four segmentation bases that can be utilised to identify groups to inform programs development and implementation. Segmentation in social marketing offers a meaningful opportunity for future research to extend market insights and the use of all four segmentation bases arguably is expected to help enhance behaviour change.

Insights gained indicate group differences which can be used to guide medical service decisions and behavioural change program planning and design. Segment one reported risky sexual practices with rates of risky behaviours, sexual activity, STI testing and STI treatment far higher than segments two and three. While differences are evident that can be used to inform program planning and design there are commonalities that deserve attention throughout the broader population. Insights gained in the current study indicate that there is a lack of knowledge and understanding on the topic of sexual health and related behaviours. Medical service provision, public health, education and other behavioural change fields need to be engaged to overcome misperceptions and knowledge deficits.

From a social marketing standpoint segment one may warrant targeting with a targeted condom use program focussed on making condom use more fun. Innovative and fun delivery methods could be considered. For example, a social media approach could be used to promote condom use through engagement with the target audience. With the increasing use of the Internet and mobile devices the promotion of sexual health programs through digital media offers large potential in a young adult population. Digital media can be customised for specific populations and communities and it gives an opportunity for self-directed learning through private use [73].

### Limitations and future research

Taken together, this study aimed to investigate the applicability of segmentation within the context of improving the safe sex behaviour of young people in Queensland, Australia. The results indicated three distinct segments in a sexual health context. Respondents within these segments had significantly different sexual behaviours, STI testing and treatment behaviours, demographic, geographic and psychographic profiles. However, the generalisability of the results to a broader Australian context is limited given this study used a convenience, cross-sectional sample limited to one Australian state. It is recommended to implement a larger national or international scale study to extend understanding and to permit generalisability of results.

A methodological limitation arising in this study centred on the items used to explore sexual behaviour in 15–29 year olds. Some lines of questioning were not applicable for some or many respondents (e.g. respondents who aren’t sexually active) and question structure resulted in high levels of missing data in some lines of questioning. Missing data is not treated in two-step cluster analysis and as such some measures could not be used in segmentation analysis in the current study. Improved question framing to ensure items are relevant to all respondents and pilot testing of developed surveys is recommended in future studies permitting all measures to qualify for segmentation analysis.

Additionally, it was not examined if the identified segments in this study respond to a sexual health social marketing program differently. Use of a longitudinal research design is recommended to enable an examination of response for each of the identified segments in this study to a social marketing intervention targeting safe sex behaviour for young people. This study provides a basis for future research examining the applicability and usefulness of all four segmentation bases in segmentation approaches aiming to change the safe sex behaviour of young people.

The current study is not theoretically informed. The inclusion of theory in social marketing is recommended as a social marketing benchmark practice [74] and this represents an additional opportunity for future research. Research indicates that when theory is used in social marketing practice [75, 76] the extent of theory use is limited [77]. Recent examples showcasing the use of the Theory of Interpersonal Behaviour and the Theory of Planned Behaviour in conjunction with two-step cluster analysis are available [50, 78]. Luca & Suggs and Truong et al. [75, 76] highlight commonly used theories in social marketing. Future research is recommended to test the array of theories to understand their ability to identify segments and importantly in time increase behavioural change outcomes.

The predictive capability of demographic and geographic variables to predict behaviour has been challenged elsewhere [43, 45]. Future longitudinal research is recommended to examine the predictive capacity of each of the four segment bases and on behavioural change. Understanding which variables and bases are associated with behavioural change can inform segmentation practice. In addition, field trials that apply segment driven insights may deliver further empirical evidence to assist social marketing to understand which approach delivers optimal outcomes.

## Conclusions

This study examined young people aged 15–29 in Queensland, Australia to gain segment level insights to demonstrate the potential for segments derived from four bases to be combined with available data for the purposes of informing social marketing program design. Social marketers can measure variables across all four segmentation bases to identify smaller homogenous segments within a larger heterogeneous target audience. Insights gained can be used to deliver a dashboard that in turn can be used to generate specific programs to target the wants and needs of each group, which in turn is expected to enhance behavioural change [28, 29]. This study highlighted the importance of behavioural variables in segmentation, given the importance of these variables in segment definition, comprehension and understanding.

## Additional file

Additional file 1: Online survey. Queensland Health Sexual Health Formative Research 2017 Quantitative Questionnaire. Survey with Queensland residents about their sexual health and wellbeing. (PDF 523 kb)

## Abbreviation

STI: Sexually Transmissible Infection
