# Supplementary material for: Learning what our target audiences think and do: extending segmentation to all four bases
Source: BMC Public Health. 2019 Apr 5;19:382. doi: 10.1186/s12889-019-6696-2 (PMC6451251; doi:10.1186/s12889-019-6696-2)
Supplement: Supplementary file 1 — Online survey. Queensland Health Sexual Health Formative Research 2017 Quantitative Questionnaire. Survey with Queensland residents about their sexual health and wellbeing. (PDF 523 kb) [file 12889_2019_6696_MOESM1_ESM.pdf]

Queensland Health
Sexual Health Formative Research 2017
Quantitative Questionnaire
FINAL
June 2017

Introduction:

We have been commissioned on behalf of Enhance Research to undertake a survey with Queensland residents on behalf of the Queensland Government about their **sexual health and wellbeing**.

Introduction for alternate sample sources:
Enhance Research is currently conducting an interesting new survey with Queensland residents on behalf of the Queensland Government about their **sexual health and wellbeing**.

*The survey will take **approximately 20 minutes** to complete depending on your answers.*

FOR ALL

PLEASE NOTE: While the topic of sexual health and wellbeing is of a personal nature, we would encourage you to be as frank and honest in your answers as possible; we'd really like to know your own views. All your answers are confidential and private; if you suspect you may become upset by answering questions about your sexual health, we recommend you do not take part in this research study.

The research is covered by Market and Social Research Privacy Principles with findings from the surveys reported only in aggregate, so your responses and opinions will not be identified to the Queensland Government.

Please do not use the back and forward buttons in the browser. Instead, please use the "Next" and "Back" buttons at the bottom of each page.

This survey has received ethical clearance from Griffith University. You indicate your consent to participate in the study by completing the survey.
For more information please click here.
(INCLUDE BACK BUTTON)

SCREENING:

S1. How old are you?

Under 15 (screen out)	❍ 1
15	❍ 2
16	❍ 3
17	❍ 4
18	❍ 5
19	❍ 6
20	❍ 7
21	❍ 8
22	❍ 9
23	❍ 10
24	❍ 11
25	❍ 12
26	❍ 13
27	❍ 14
28	❍ 15
29	❍ 16
30 or over (screen out)	❍ 17

S2. Which state do you live in?

Queensland	❍ 1	
NSW	❍ 2	TERMINATE
Victoria	❍ 3	
ACT	❍ 4	
SA	❍ 5	
TAS	❍ 6	
WA	❍ 7	
NT	❍ 8	

S3. Are you…

Male	❍ 1
Female	❍ 2
Gender diverse	❍ 3
Intersex	❍ 4
Other	❍ 5

S4. Are you? SINGLE RESPONSE

Aboriginal	❍1
Torres Strait Islander	❍2
Aboriginal and Torres Strait Islander	❍3
Neither	❍4

S5. Do you consider yourself to be? SINGLE RESPONSE

Straight	❍1
Gay male	❍2
Lesbian	❍3
Bisexual	❍4
Queer	❍5
Other (Specify)	❍6

S6. Were you born in a non-English speaking country? SINGLE RESPONSE

Yes	❍1
No	❍2

S7 Were either of your parents born in a non-English speaking country? SINGLE RESPONSE

Yes	❍1
No	❍2

SECTION 1: KNOWLEDGE & UNDERSTANDING

The following questions are about what **'sexual health'** means to you.

Q2. Which of the following do you consider when thinking about sexual health?

RANDOMISE	X
Having sex	❑1
Emotional, mental and social wellbeing	❑2
Respectful interactions and relationships	❑3
Sexually transmissible infections (STIs)	❑4
Sexuality	❑5
Sexual preference	❑7
Expression of intimacy	❑8
Communication between sexual partners	❑10
Consent (i.e. agreeing to sexual activity with someone)	❑11
Number of partners	❑12
Using protection	❑15
Regular STI testing/screening	❑16
Birth control methods	❑17
Practicing safe sex	❑18
Sexual assault	❑19
Sexual coercion	❑20
Sexual pleasure	❑21
Fulfillment	❑22
Abstinence	❑23
Other (specify)	❑99
Don't know	❍98

Q3. How much do you agree with the following statements about **sexual health**?

**RANDOMISE**	Strongly disagree						Strongly agree					DK
Sexual health is an important topic to me	0	1	2	3	4	5	6	7	8	9	10	98
I have a good understanding of sexual health	0	1	2	3	4	5	6	7	8	9	10	98
Sexual health education should be provided in schools	0	1	2	3	4	5	6	7	8	9	10	98
Schools and parents should share responsibility for providing sexual health education	0	1	2	3	4	5	6	7	8	9	10	98
The sexual health education I received in school covered the topics that I need	0	1	2	3	4	5	6	7	8	9	10	98
There are certain topics which should not be discussed outside of one's immediate family, sexual health is one of these	0	1	2	3	4	5	6	7	8	9	10	98
My parents help me to understand things that relate to my sexual health	0	1	2	3	4	5	6	7	8	9	10	98
My friends help me to understand things that relate to my sexual health	0	1	2	3	4	5	6	7	8	9	10	98

Q4A. What are the sources of information and education about sexual health that you have used?

SHOW SELECTED AT Q4a

Q4B. Of these, what is your main source of information and education about sexual health?

Q4C. If you could choose, which of these would you prefer to be your main source of information and education about sexual health?

	**Q4A**	**Q4B**	**RECODE from**	**Q4C**
Parents	❑1	❍1	❍1	❍1
Other family members	❑2	❍2	❍2	❍2
Siblings – brothers or sisters	❑3	❍3	❍3	❍3
School class	❑4	❍4	❍4	❍4
Friends and peers	❑5	❍5	❍5	❍5
Magazines	❑7	❍7	❍6	❍7
Internet searches	❑8	❍8	❍7	❍8

Specific websites (specify)	❑10	❍10	❍8	❍10
GPs (doctor)	❑11	❍11	❍9	❍11
Sexual health clinics	❑12	❍12	❍10	❍12
Support groups and organisations (specify)	❑15	❍15	❍11	❍15
True (formerly Family Planning)	❑16	❍16	❍12	❍16
Porn sites	❑17	❍17	❍13	❍17
Books	❑18	❍18	❍14	❍18
Other (specify)	❑99	❍99	❍16	❍99
Don't know	❍98			
Sexual partners	❑19	❍19	❍15	❍19

Q5. Are you familiar with the term **sexually transmissible infections (STIs)**? SINGLE RESPONSE

Yes, I'm familiar with the term	❍1
Yes, I've heard of it, but not really familiar	❍2
No, not heard of it	❍3

ASK IF YES TO Q5, ELSE SKIP TO Q9

Q6. What does the term **sexually transmissible infections (STIs)** mean to you? OPEN ENDED

ASK IF Q5=1,2

Q7. Can you name all of the **sexually transmissible infections (STIs)** that you are aware of? OPEN ENDED

ASK IF Q5=1,2

Q8. How would you rate your knowledge when it comes to the following STI's?

RANDOMISE	**Never heard of it**	**I know a little about it**	**I know a lot about it**
Chlamydia	❑1	❑1	❑1
Syphilis	❑2	❑2	❑2

Gonorrhoea	❑3	❑3	❑3
HIV	❑4	❑4	❑4
Genital Herpes	❑5	❑5	❑5
HPV (human papillomavirus)	❑7	❑7	❑7
Genital Warts	❑8	❑8	❑8
Pubic lice	❑9	❑9	❑9

Q9. How much do you agree with the following statements about **STIs**?

RANDOMISE	Strongly disagree									Strongly agree		Not Applicable / Don't know
Most STIs are incurable	0	1	2	3	4	5	6	7	8	9	10	98
I have a good understanding of STIs	0	1	2	3	4	5	6	7	8	9	10	98
I feel I might be at risk of an STI because of my sexual activity	0	1	2	3	4	5	6	7	8	9	10	98
STIs are a problem in society	0	1	2	3	4	5	6	7	8	9	10	98
Using condoms is the best protection against STIs	0	1	2	3	4	5	6	7	8	9	10	98
I am conscious of protecting myself against STIs when I have sex	0	1	2	3	4	5	6	7	8	9	10	98
I am conscious of protecting myself against STIs during oral sex	0	1	2	3	4	5	6	7	8	9	10	98
There are other ways (other than condoms) to protect against STIs	0	1	2	3	4	5	6	7	8	9	10	98
ASK IF S5=4 (LESBIAN)<br>Using dental dams is the best protection against STIs	0	1	2	3	4	5	6	7	8	9	10	98

Q9A. How much do you agree with the following statements about **condoms and STIs**?

RANDOMISE												
I feel confident discussing condom usage with a partner	0	1	2	3	4	5	6	7	8	9	10	98
Using a condom can be fun	0	1	2	3	4	5	6	7	8	9	10	98
I am confident I could persuade a partner to use a condom during sex	0	1	2	3	4	5	6	7	8	9	10	98

I am confident that I would use a condom during sex even after drinking alcohol	0	1	2	3	4	5	6	7	8	9	10	98
I would insist on using condoms during sex, even if my partner didn't want to	0	1	2	3	4	5	6	7	8	9	10	98
When things are getting intense, I could still make sure we use a condom	0	1	2	3	4	5	6	7	8	9	10	98
I would use a condom during sex even if it was less fun	0	1	2	3	4	5	6	7	8	9	10	98
I would use condoms during sex to avoid getting STIs	0	1	2	3	4	5	6	7	8	9	10	98
I am confident that I would use a condom even if I was high	0	1	2	3	4	5	6	7	8	9	10	98
I feel confident I could purchase condoms without feeling embarrassed	0	1	2	3	4	5	6	7	8	9	10	98

Q10. How would you rate your understanding around the following issues related to the STIs you are most aware of?

	No Understanding						Excellent Understanding					DK
Transmission – how you can get the STI	0	1	2	3	4	5	6	7	8	9	10	98
Symptoms – the symptoms of the STI	0	1	2	3	4	5	6	7	8	9	10	98
Testing – how to find out if you have the STI	0	1	2	3	4	5	6	7	8	9	10	98
Prognosis – how serious the STI can be for your health	0	1	2	3	4	5	6	7	8	9	10	98
Treatment – how to treat the STI if you have it	0	1	2	3	4	5	6	7	8	9	10	98
Prevention/contact tracing – testing and treatment of partners	0	1	2	3	4	5	6	7	8	9	10	98

Q11. What does the term **sexually active** mean to you? Is this someone who..

RANDOMISE	**Q11**
Has had sexual intercourse with someone (vaginal or anal penetration)	❑1
Has had oral sex with someone	❑2
Has masturbated with someone	❑3
Has kissed someone	❑4

Masturbates alone	❑5
Uses sex toys	❑7
Other (specify)	❑99
Don't know	❍98

SECTION 2: BEHAVIOUR

The next set of questions relate to the things that you do when it comes to sex and your sexual health.

Q12. Which of the following have you **ever** done?
IF Code 97, skip Q12A, Show responses from Q12
Q12A. Which of these have you done in the past three months?

	Q12	Q12A
Had sex (vaginal)	❑1	❑1
Had sex (anal)	❑2	❑2
Had oral sex (given or received a blow job)	❑3	❑3
Had oral sex (gone down on someone / someone gone down on you)	❑4	❑4
Had anonymous sex	❑5	❑5
Had casual sex	❑7	❑7
Been tested for STIs	❑8	❑8
Been treated for STIs	❑10	❑10
Had group sex (threesome or more)	❑11	❑11
Had sex while drunk	❑12	❑12
Had sex while high	❑13	❑13
Had sex while drunk & high	❑14	❑14
Been coerced into having sex (when you didn't want to)	❑15	❑15
Had sex with someone you had found through hook-up apps (e.g. TINDER, GRINDR)	❑16	❑16
Had a sexual experience where consent was withdrawn or changed during sexual activity (by you or your partner)	❑17	❑17
Regretted having sex with someone	❑18	❑18
Other (specify)	❑99	❑99
None of these	❍97	❍97

MID-SURVEY REFERRAL:

Thanks for your responses so far, we still have more questions to ask you but if this survey has raised any issues or concerns for you about your sexual or general health and wellbeing there are a number of organisations that you can contact for further support or information.

Lifeline 13 11 14
Kids Helpline 1800 55 1800
National Sexual Assault, Family & Domestic Violence Counselling Line 1800 RESPECT (1800 737 728)
Queensland Statewide Sexual Assault Helpline 1800 010 120

Alcohol and Drug Information Service 1800 177 833 (ADIS provides a free, 24 hour/7 day, counselling, information and referral service for anyone with concerns about their own or someone else's use of alcohol or other drugs. This is an anonymous and confidential service).
Open Doors 3257 7660
Police Link 131 444
For STI testing and treatment services - https://www.health.qld.gov.au/clinical-practice/guidelines-procedures/sex-health/services

ASK IF YES TO Q12 1-4 IF NO GO TO Q21
Q13. How many sexual partners have you had in the past three months? SINGLE RESPONSE

No partners	❍1
1 partner	❍2
2 partners	❍3
3 partners	❍4
4 or more partners	❍5

ASK IF YES TO Q12 1-4 IF NO GO TO Q21
Q14. Are you in a monogamous relationship (both of you have each other as your only sexual partner)? SINGLE RESPONSE

Yes	❍1
No	❍2
Not sure	❍3

ASK ALL
Q15. Have you ever heard of any of the following approaches to provide protection against STIs when having sex? MULTIPLE RESPONSE

ASK IF YES TO Q12 1-4 IF NO GO TO Q21
Q15A. When you have sex have you used? MULTIPLE RESPONSE

	**Q15**	**Q15A**
Condoms	❑1	❑1
Oral contraceptives (the pill)	❑2	❑2
Withdrawal method (pulling out)	❑3	❑3
HIV PrEP (Pre Exposure Prophylaxis)	❑4	❑4
HIV PEP (Post Exposure Prophylaxis)	❑5	❑5
IUDs (Inter Uterine Device)	❑7	❑7
Dental dams	❑8	❑8
Washed/cleaned sex toys	❑10	❑10
Other (specify)	❑99	❑99
Don't know	❍98	

None of these	[redacted]	❍97

ASK FOR Q15A = 1 &/OR 8 CONDOM USE &/OR DAM USE

Q16. How frequently would you say you use condoms or dams? SINGLE RESPONSE

Every time	❍1
Most of the time	❍2
Half the time	❍3
Some of the time	❍4
Hardly ever	❍5
Only used once	❍6

ASK IF Q15A = 1 CONDOM USE

Q17. What are the reasons for you using a condom? MULTIPLE RESPONSE

(IF ONE RESPONSE AT Q17, AUTO-CODE INTO Q17A.

Q17A. What is the key reason for you using a condom? SINGLE RESPONSE

	**Q17**	**Q17A**
Contraception (to prevent pregnancy)	❑1	❍1
Prevention of STIs	❑2	❍2
My partner insisted	❑3	❍3
None of the above	❑4	❍4

ASK IF Q15A=1 CONDOM USE

Q18. Did you use a condom the last time you had ? SINGLE RESPONSE

	Yes	No
ASK IF Q12 = 1 Vaginal sex	❍1	❍2
ASK IF Q12 = 2 Anal sex	❍1	❍2
ASK IF Q12 = 3 Oral sex	❍1	❍2

ASK IF YES TO Q12 1-4

Q19. Have you ever? MULTIPLE RESPONSE

	Yes	No
Asked a partner to use a condom	❍1	❍2
Discussed condom use with a partner	❍1	❍2

ASK IF Q19=YES

Q20. How often do you talk about condom usage with a new partner/s ? SINGLE RESPONSE

	**Q20**
Every time	❍1
Most of the time	❍2
Half the time	❍3
Some of the time	❍4
Hardly ever	❍5
Only once	❍6

ASK ALL

Q21. Thinking about your friends and peers, how much do you agree with the following statements?

RANDOMISE	Strongly disagree						Strongly agree					DK
My friends always use condoms when having sex with new partners	0	1	2	3	4	5	6	7	8	9	10	98
My friends think it is important to use a condom when having sex with a new partner	0	1	2	3	4	5	6	7	8	9	10	98
Most of my friends think you should avoid unsafe sex	0	1	2	3	4	5	6	7	8	9	10	98
Most people I know have safe sex all the time	0	1	2	3	4	5	6	7	8	9	10	98
My friends and I discuss sex with one another	0	1	2	3	4	5	6	7	8	9	10	98

ASK IF Q12 = 12,13,14 (DRUNK &/OR HIGH)

Q22a. How much do you agree with the following statements?

RANDOMISE	Strongly disagree						Strongly agree					DK
After drinking alcohol…												
I am more likely to have sex	0	1	2	3	4	5	6	7	8	9	10	98
Sex is more pleasurable	0	1	2	3	4	5	6	7	8	9	10	98
I always use condoms when having sex	0	1	2	3	4	5	6	7	8	9	10	98
I am more likely to have oral sex	0	1	2	3	4	5	6	7	8	9	10	98

I am more likely to have anal sex	0	1	2	3	4	5	6	7	8	9	10	98
I am more likely to have group sex	0	1	2	3	4	5	6	7	8	9	10	98
Q22b. After using drugs…												
Sex is more pleasurable	0	1	2	3	4	5	6	7	8	9	10	98
I am more likely to have sex	0	1	2	3	4	5	6	7	8	9	10	98
I always use condoms when having sex	0	1	2	3	4	5	6	7	8	9	10	98
I am more likely to have oral sex	0	1	2	3	4	5	6	7	8	9	10	98
I am more likely to have anal sex	0	1	2	3	4	5	6	7	8	9	10	98
I am more likely to have group sex	0	1	2	3	4	5	6	7	8	9	10	98

SECTION 3: CLINICAL EXPERIENCE & ATTITUDES

The next set of questions relate to your experiences with health professionals about your sexual health. By health professionals we mean doctors or GPs, nurses and other staff in clinics.

ASK ALL
Q23. Have you ever discussed sexual health or had an STI test with a doctor, nurse or other health professional? SINGLE RESPONSE

Yes – discussed sexual health but never tested for an STI	❍1
Yes – been tested for an STI	❍2
Yes – both discussed sexual health and tested for an STI	❍3
No -neither	❍4

ASK IF Q23 = 2 OR 3 (TESTED)
Q24. How recently was your most recent STI test? SINGLE RESPONSE

Within the past month	❍1
Within the past three months	❍2
Within the past year	❍3
More than one year ago	❍4

ASK IF Q23 = 2 OR 3 (TESTED)
Q25. What motivated you to have your most recent STI test? MULTIPLE RESPONSE

I test regularly	❑ 1
I experienced some symptoms that I thought I should get checked	❑ 2
I had unprotected sex	❑ 3
I experienced a penile, vaginal or anal injury during sex	❑ 4
I thought it was something I should do	❑ 5
Other (please specify)	❍ 99

ASK IF Q23 = 2 OR 3 (TESTED)

Q26. Where did you have your most recent STI test? SINGLE RESPONSE

GP (your regular doctor)	❍ 1	❍ 1
GP (other than your regular doctor)	❍ 2	❍ 2
Sexual health clinic	❍ 3	❍ 3
True (formerly Family Planning)	❍ 4	❍ 4
Aboriginal Medical Service	❍ 5	❍ 5
Hospital	❍ 7	❍ 6
Other (please specify)	❍ 99	❍ 7

ASK IF Q23 = 1,2 OR 3 (DISCUSSED &/OR TESTED)

Q27. Thinking about your experience with the health professionals you have dealt with in relation to your sexual health, how much do you agree with the following statements?

**RANDOMISE**	Strongly disagree						Strongly agree					DK
I am satisfied with the sexual health services that I receive from staff	0	1	2	3	4	5	6	7	8	9	10	98
I get support from staff for the challenges of being sexually active	0	1	2	3	4	5	6	7	8	9	10	98
My main healthcare provider assumes I always use condoms	0	1	2	3	4	5	6	7	8	9	10	98
My main healthcare provider provides information, fliers and pamphlets about STIs and STI transmission that are easy to understand	0	1	2	3	4	5	6	7	8	9	10	98

ASK ALL

Q28. Thinking about your expectations of health professionals generally in relation to sexual health, how much do you agree with the following statements?

RANDOMISE	Strongly disagree						Strongly agree					DK
Health professionals are generally approachable to discuss sexual health	0	1	2	3	4	5	6	7	8	9	10	98
I am comfortable discussing my sexual health with a health professional	0	1	2	3	4	5	6	7	8	9	10	98
Health professionals are understanding and empathetic about sexual health	0	1	2	3	4	5	6	7	8	9	10	98
Health professionals understand the issues facing young people who are sexually active	0	1	2	3	4	5	6	7	8	9	10	98
I find it embarrassing to discuss sexual health with a health professional	0	1	2	3	4	5	6	7	8	9	10	98
I would rather not discuss sexual health with health professionals because it is a private matter	0	1	2	3	4	5	6	7	8	9	10	98
Health professionals are judgemental and make me feel ashamed about sexual health	0	1	2	3	4	5	6	7	8	9	10	98

ASK IF S4=1,2,3 ABORIGINAL &/OR TORRES STRAIT ISLANDER

Health professionals understand my needs as an Aboriginal or Torres Strait Islander person	0	1	2	3	4	5	6	7	8	9	10	98

ASK IF S3=3,4,5 GENDER DIVERSE, INTERSEX OTHER

Health professionals understand my needs as a Gender Diverse person	0	1	2	3	4	5	6	7	8	9	10	98

ASK IF S5=2,3,4,5 GAY, LESBIAN, BISEXUAL, QUEER

Health professionals understand my needs as a Gay or Lesbian or Bisexual or Queer person	0	1	2	3	4	5	6	7	8	9	10	98

ASK IF S6 OR S7 = YES

Health professionals understand my needs as someone from a non-English speaking background	0	1	2	3	4	5	6	7	8	9	10	98

ASK ALL

Q29. Thinking about dealing with health professionals about sexual health, which of the following would you choose? SINGLE RESPONSE FOR EACH

I would prefer to deal with a health professional…

I did not know	❍1
I already know	❍2

Who focuses on providing positive advice about being sexually active	❍1
Who focuses on outlining the risks associated with being sexually active	❍2

ASK ALL

Q30. When thinking about discussing sexual health with a health professional which of these emotions do you feel? MULTIPLE RESPONSE **RANDOMISE**

Anger	❑1
Contempt	❑2
Disgust	❑3
Distress	❑4
Fear	❑5
Guilt	❑6
Interest	❑7
Joy	❑8
Shame	❑9
Surprise	❑10
Shy	❑11
Nervous	❑12
Normal	❑13
Embarrassed	❑14
None of these	❍99

SECTION 4: OVERALL ATTITUDES

We appreciate you sticking with us. We're nearly at the end!
Now we'd like to understand your general attitudes towards sexual health.

ASK ALL
Q31. Thinking about sexual health generally, how much do you agree with the following statements?

**RANDOMISE**	Strongly disagree						Strongly agree					Don't know / Not applicable
I am comfortable discussing sexual health issues with members of my family	0	1	2	3	4	5	6	7	8	9	10	98
I am comfortable discussing sexual health issues with my friends	0	1	2	3	4	5	6	7	8	9	10	98
I feel confident in my ability to form respectful sexual relationships	0	1	2	3	4	5	6	7	8	9	10	98
I feel comfortable with my sexuality	0	1	2	3	4	5	6	7	8	9	10	98
I see sex as an expression of intimacy	0	1	2	3	4	5	6	7	8	9	10	98
I enjoy sex	0	1	2	3	4	5	6	7	8	9	10	98
I have had sexual experiences that I was not comfortable with	0	1	2	3	4	5	6	7	8	9	10	98
I am careful about choosing who I have sex with	0	1	2	3	4	5	6	7	8	9	10	98
I feel I have little control over my sexual health	0	1	2	3	4	5	6	7	8	9	10	98
Alcohol has a negative impact on my sexual health	0	1	2	3	4	5	6	7	8	9	10	98
Drugs have a negative impact on my sexual health	0	1	2	3	4	5	6	7	8	9	10	98

DEMOGRAPHICS:

And to finish up, we'd like to ask you a few general questions about yourself.

D1. Which of the following best describes you? MULTIPLE RESPONSE

Studying at school	❑1
Studying or training at Vocational Education or Training college (e.g. TAFE, apprenticeship)	❑2
Studying at university	❑3
Employed full time	❑4
Employed part time or casual	❑5
Not employed	❑6
Other	❑7

ASK IF D1 = 4,5,6 (EMPLOYED OR NOT EMPLOYED) AND D1≠1,2,3 (STUDYING)

D2. What is your highest level of education? SINGLE RESPONSE

Primary school	❍1
Some high school	❍2
Year 10	❍3
Year 12	❍4
TAFE or college	❍5
Undergraduate degree	❍6
Post-graduate degree	❍7

D3. What is your postcode (or if not known please enter your suburb or town)?

____

D4. Are you a parent? SINGLE RESPONSE

Yes	❍1
No	❍2
Pregnant	❍3

D5. Which of the following best describes your household? SINGLE RESPONSE

I live at home with my parent/s	❍1
I live alone	❍2
I live in a shared household	❍3

I live with my partner	❍4
I am a single parent	❍5
I am in a couple with children at home	❍6
I live in shared student accommodation (eg on campus)	❍7
Other (specify)	❍99

D6. Do you consider yourself as someone living with a disability? SINGLE RESPONSE

Yes	❍1
No	❍2

D7. Do you speak a language other than English at home? SINGLE RESPONSE

Yes	❍1
No	❍2

ASK IF D7=YES LANGUAGE

D8. Other than English, what language/s do you speak? OPEN ENDED

THANK YOU, THAT IS THE END OF THE SURVEY

END OF SURVEY REFERRAL:

If this survey has raised any issues or concerns for you about your sexual or general health and wellbeing there are a number of organisations that you can contact for further support or information.

LIST SUPPORT LINKS
